# Consistent condom use and its associated factors among human immunodeficiency virus-positive pregnant women in Ethiopia

**DOI:** 10.3389/fmed.2022.907879

**Published:** 2022-08-04

**Authors:** Nebiyu Solomon Tibebu, Belayneh Ayanaw Kassie, Azmeraw Ambachew Kebede, Tazeb Alemu Anteneh, Wubedle Zelalem Temesgan, Mastewal Belayneh Aklil, Kindu Yinges Wondie, Marta Yimam Abegaz, Hiwotie Getaneh Ayalew, Bayew Kelkay Rade

**Affiliations:** ^1^Department of Clinical Midwifery, School of Midwifery, College of Medicine and Health Sciences, University of Gondar, Gondar, Ethiopia; ^2^Department of Women’s and Family Health, School of Midwifery, College of Medicine and Health Sciences, University of Gondar, Gondar, Ethiopia; ^3^Department of Midwifery, School of Nursing and Midwifery, College of Medicine and Health Sciences, Wollo University, Dessie, Ethiopia; ^4^Department of General Midwifery, School of Midwifery, College of Medicine and Health Sciences, University of Gondar, Gondar, Ethiopia

**Keywords:** condom, HIV, pregnant, women, Ethiopia

## Abstract

**Background:**

Consistent condom use plays a significant role in the successful protection of the transmission of human immunodeficiency virus (HIV) infection in couples with sero-discordant HIV status, mother-to-child-transmission (MTCT), and acquiring other strains in HIV-positive concordant pairs. Limited data and information about this issue are available in low-resource setting countries, including the study setting. Therefore, this study aimed to assess the level of consistent condom use and its associated factors among HIV-positive pregnant women.

**Materials and methods:**

An institution-based cross-sectional study was done from 17 October 2020 to 1 March 2021. A total of 423 HIV-positive pregnant women were involved in this study and selected using a systematic random sampling technique. Data were collected using a semi-structured, pretested, and interviewer-administered questionnaire and then entered into EPI INFO version 7 and analyzed using Statistical Package for Social Sciences (SPSS) version 21. Logistic regressions were performed to identify factors. *P*-Value ≤ 0.05 denotes statistical significance.

**Results:**

The prevalence of consistent condom use among HIV-positive pregnant women was 14.2% [95% confidence interval (CI) 10.9%, 17.5%]. Women having a higher educational status [adjusted odds ratio (AOR) = 6.33, 95% CI 1.96, 20.42], women having a CD4 count > 600 cells/mm (AOR = 4.78, 95% CI 2.08, 10.97), women testing positive for HIV during the non-pregnant state (AOR = 5.99, 95% CI 3.01, 11.94), and women disclosing their HIV status to sexual partners (AOR = 4.85, 95% CI 1.71, 13.71) were found to be statically significant with women’s consistent condom use.

**Conclusion:**

In this study, consistent condom use among HIV-positive pregnant women was low. Women having educational status of college and above studies, women testing positive for HIV during the non-pregnant state, women disclosing their HIV status to a sexual partner, and women having a CD4 count > 600 cells/mm had better consistent condom use. Hence, giving more emphasis on health education and counseling service about HIV testing before pregnancy, and disclosing their HIV status to their sexual partners and about the need for consistent condom use during pregnancy would be important.

## Introduction

Human immune virus/acquired immune deficiency syndrome (HIV/AIDS) is a major public health concern that affects millions of people globally. Almost 37.6 million people across the world live with HIV/AIDS (1). Of these, approximately 25.7 million people (68.4%) live in Africa, among which 12 million were women (1–3). This value is also higher in Ethiopia, where an estimated 650,000 (63.08%) women were infected with the virus in 2018 (4). A study showed ∼5.74% HIV-positive pregnant women in Ethiopia (5).

Many HIV-positive pregnant women lack access to HIV prevention, treatment, and sexual and reproductive healthcare services. Similarly, children continue to become infected during the perinatal stage (6). This results in the loss of lives of many women and children due to poor prevention and control of HIV infections (6, 7). The prevention and control of new infections among pregnant women are enhancing the women’s as well as the children’s health (6, 8).

Consistent condom use among HIV-positive pregnant women is one of the four cornerstones of a comprehensive program for the prevention of mother-to-child HIV transmission (PMTCT) and reduces the chances of getting new drug-resistant viral strains (7, 9, 10). It also reduces the risk of new infections transmission among sero-discordant couples. Consistent use of condom could reduce the transmission of HIV infection by > 80% (11). In addition, it plays a significant role in preventing other sexually transmitted diseases (STDs) (12, 13). The presence of STD can increase the risk of mother-to-child HIV transmission and leads to adverse birth outcomes among HIV-infected women (14, 15). However, numerous studies have shown that the level of barrier method utilization among HIV-positive people in the developing countries is low (7, 9, 10).

The level of condom use is distinct in different countries. The prevalence of consistent condom use among HIV-positive pregnant women was 26.2% in Nigeria (7) and 48% in Brazil (16). The factors associated with inconsistent condom use were cost, religion perception, alcohol use, perception of diminished sexual satisfaction, low perception risk of STDs, condom fatigue, having a stable sexual partner and emotional fulfillment, mistrust in relationships, gender inequality, perceptions of modesty, and low condom self-efficacy (17–19). Other studies show that self-efficacy is the most important conjecturer of condom use (20–22).

Controlling new HIV infection is one of the principal health priorities of the Federal Ministry of Health of Ethiopia. To achieve this, the government of Ethiopia designed and implemented various approaches to prevent HIV transmission by increasing the coverage of antiretroviral therapy (ART) and national condom distribution program. Condoms can be distributed in the following ways: deliver condoms free of charge, cover widescale distribution, implement social marketing campaigns to encourage condom use, distribute to individuals and organizations, and establish organizational support for condom distribution (23, 24). However, the nature of the epidemic is a challenge to meet the target for HIV/AIDS prevention and control in the country (25). Despite these interventions, the issue still continues to be a public health problem, and there is a paucity of evidence that has assessed the prevalence and factors affecting consistent condom use among HIV-positive pregnant women in Ethiopia. Therefore, this study aimed to assess the level of consistent condom use and associated factors among HIV-positive pregnant women who attended prenatal care in the Amhara regional state referral hospitals.

## Materials and methods

### Study design, area, and period

An institution-based cross-sectional study was conducted from 17 October 2020 to 1 March 2021. The study was conducted in the Amhara Region Referral Hospitals, Ethiopia, namely, the University of Gondar (UoG) Comprehensive Specialized Hospital, Felege Hiwot Comprehensive Specialized Hospital, Tibebe Giyon Comprehensive Specialized Hospital, Debremarkos Comprehensive Specialized Hospital, Dessie Comprehensive Specialized Hospital, and Debrebirhan Comprehensive Specialized Referral Hospital. The Amhara region is the second largest and most populous regional state in Ethiopia. The prevalence of HIV among adults and pregnant women in this region is 1.5 and 0.8%, respectively (26).

### Study population

The study population included all HIV-positive pregnant women who attended antenatal care (ANC) follow-up in the Amhara Region Referral Hospitals during the study period.

### Eligibility criteria

Human immunodeficiency virus -positive pregnant women who attended ANC at the time of the study and those aged ≥ 18 years were included. Women knew their HIV status during data collection, and those who were mentally ill and unable to communicate verbally were excluded from this study.

### Sample size determination and sampling procedure

The sample size was determined using a single population proportion formula using the formula of $n=\frac{{\left(Z\,\alpha /2\right)}^{2}*p\,\left(1-p\right)}{d^{2}}=\frac{{\left(1.96\right)}^{2}*0.5\,\left(1-0.5\right)}{\left(0.05\right)\,2}=384$, where n is the required sample size, α is the level of significant, *z* is the standard normal distribution curve value for 95% confidence level = 1.96, *p* is the proportion, and *d* is the margin of error. Taking 50% proportion, since there is no study with 95% level of confidence, and 5% margin of error and considering a 10% non-response rate, the final sample size was 423. Using the PMTCT registration book of each hospital, the sample size was dispersed proportionally to the respective hospitals, and then the samples were selected at intervals of two, which was systematic random sampling method.

### Operational definitions and measurements

#### Consistent condom use

This is defined as “always” using condoms with all sexual partners during every sexual intercourse since HIV diagnosis after the conception of the current pregnancy (16).

#### Having good knowledge

A woman who responds to 3–5 of the HIV knowledge questions correctly (27).

Consistent condom use was the dependent variable for this study and was measured using a dichotomized response (yes/no) question that is consistent with an earlier study (28). The questions were “Did you or your sexual partner use condom during sexual intercourse after conception of the current pregnancy? Used for endorsement purposes.” If the answer was “yes,” “Did you always use a condom?” was the next question to determine whether the use was consistent or not.

### Independent variables

Age of the women, residence, educational status of women and husband, household income, women’s occupation, religion, marital status, discussing about HIV with a sexual partner, partner’s HIV status, number of pregnancies, number of alive children, pregnancy status, ANC visit, time since HIV diagnosis, duration of known positive for HIV, being on ART treatment and duration, infected with hepatitis B virus (HBV), getting counseling before the test, getting counseling after test, getting ongoing counseling every visit, a member of an association of people living with acquired immune deficiency virus (PLWHA), infected family, disclosing HIV status to sexual partner, and knowledge on HIV transmission and prevention were the independent variables.

### Data collection tool and procedure

The data collection tool was developed by revising related studies (7, 16, 22, 25, 29). A semi-structured questionnaire was used to collect the data through face-to-face interviews. Sociodemographic, obstetrics, clinical, and knowledge on HIV-related characteristics were integrated in the study tool. Six midwives with BSc degree and four midwives with master’s degree were recruited for data collection and supervision, respectively.

### Data quality control

The questionnaire was first prepared in English and then translated to the local language (Amharic) and back to English to maintain its consistency. Before the actual data collection, a pretest was done on 5% of the sample size to check the response, language clarity, understanding of data collectors and supervisors. Training was given for a day to all data collectors and supervisors on how to approach the study subjects and collect the data, and on the objective of the study to have a better understanding of the overall process before the onset of actual data collection.

### Data processing and analysis

Data cleaning and cross-checking were done before data analysis. Data were checked, coded, and entered into EPI INFO version 7, and then exported to Statistical Package for Social Sciences (SPSS) version 21 for analysis. Both descriptive and analytic statistics were done. Binary logistic regression was done to identify statistically significant independent variables, and variables having a *p*-value of < 0.2 were entered into the multivariable logistic regression analysis for monitoring possible confounders. In the multivariable logistic regression analysis, a *p*-value of ≤ 0.05 with a 95% confidence interval (CI) for the adjusted odds ratio (AOR) was used to conclude the significance association. The final model was tested using Hosmer and Lemeshow’s goodness of fit for model fitness.

## Results

### Sociodemographic-related characteristics of study participants

A total of 423 HIV-positive pregnant women were interviewed, with a response rate of 100%. The mean age of the study participants was 29.61 years old (SD ± 5.01), and 62.2% of the participants’ age was between the age group of 25–34 years old. Two-thirds (65%) of the participants were Orthodox Christian religion followers, 80.1% of them lived in the urban setting, and 301 (71.2%) were married (Table 1).

**TABLE 1:** Sociodemographic-related characteristics of study participants in the Amhara Region Referral Hospitals, Ethiopia, 2020/2021 (*n* = 423).

Characteristics	Frequency	Percent
**Age of women in a year**		
18–24	85	20.1
25–34	264	62.4
≥ 35	74	17.5
**Residence**		
Rural	90	21.3
Urban	333	78.7
**Religion**		
Orthodox Christian	280	66.1
Muslim	119	28.1
Protestant	24	5.8
**Women’s educational status**		
Can’t read and write	56	13.2
Can read and write	73	17.3
Primary (1–8)	75	17.7
Secondary (9–12)	129	30.5
College and above	90	21.3
**Women’s occupation**		
House-wife	101	24.8
Merchant	72	17.0
Governmental employee	64	15.0
Employee of private organizations	55	13.0
NGO employee	40	9.5
Farmer	61	14.4
Others^a^	30	7.0
**Current marital status**		
Married	301	71.1
Separated and divorced	82	19.0
Single	42	9.9
**Husband educational status (*n* = 357)**		
Can’t read and write	35	9.8
Can read and write	66	18.5
Primary (1–8)	63	17.6
Secondary (9–12)	95	26.6
College and above	98	27.4
**Sexual partner’s occupation currently (*n* = 357)**		
Merchant	122	34.1
NGO employee	30	8.4
Governmental employee	72	20.2
Daily laborer	46	12.9
Farmer	43	12.0
Private organizations	44	12.4
**Average monthly income of spouses in Ethiopia (median = 2,000 ETB)**		
Median and below	349	82.5
Above median	74	17.5
**Association member of PLWHA**		
Yes	37	8.7
No	386	91.3
**Infected any family member/s**		
Yes	25	5.9
No	398	94.1

^a^Students and daily laborer.

NGO, non-governmental organization; ETB, Ethiopian Birr; PLWHIV, people living with human immune deficiency virus; HIV, human immune deficiency virus.

### Obstetrics and clinical characteristics of study participants

Of the total study participants, two-thirds (65.2%) of the participants were multigravida, and 52.7% study participants had come for their second ANC visit. Most [329 (77.8%)] participants’ pregnancy was planned. From the total study participants with HIV, 311 (73.5%) women were tested positive for HIV status during pregnancy. Approximately 309 (73.0%) study participants disclosed their HIV-positive status to their sexual partner, and 265 (62.7%) sexual partners were HIV-positive. Besides, around half of the study subjects [222 (52.5%)] had good knowledge on HIV transmission and prevention toward HIV AIDS (Table 2).

**TABLE 2:** Obstetrics and clinical and partnership characteristics of study participants in the Amhara Region Referral Hospitals, Ethiopia, 2020/2021 (*n* = 423).

Characteristics	Frequency	Percentage
**Pregnancy number**		
Prim gravida (1 pregnancy)	130	30.7
Multigravida (≥2 pregnancy)	293	69.3
**Number of alive children**		
None	154	36.4
1–5	269	63.6
**Planned pregnancy**		
Yes	329	77.8
No	94	22.2
**Duration of known positive for HIV**		
≤1 year	107	25.4
2–3 years	238	56.2
≥4 years	78	18.4
**Time of tested positive for HIV status**		
During pregnancy	311	73.5
During non-pregnant state	112	26.5
**Currently on ART**		
Yes	407	96.2
No	16	3.8
**Duration on ART (*n* = 407)**		
≤1 year	178	42.1
>1 year	229	57.9
**CD4cells/mm**		
≤600 cells/mm	206	48.7
>600 cells/mm	217	51.3
**Infected with HBV**		
Yes	17	4.0
No	406	96.0
**Counseled before test**		
Yes	225	53.2
No	198	46.8
**Counseled after test**		
Yes	399	94.3
No	24	5.7
**Got ongoing counseling every visit at the health facility**		
Yes	353	83.5
No	70	16.5
**Partner’s HIV-status**		
Negative	55	13.0
Positive	265	62.7
I do not know	103	24.3
**Disclosure HIV-status to your sexual partner (*n* = 423)**		
Yes	309	73.0
No	114	27.0
**Did you or your sexual partner use condom during sexual intercourse**		
Yes	105	24.8
No	318	75.2
**How often? (*N* = 105)**		
Always	60	14.2
Sometimes	45	10.6
**Knowledge on HIV**		
Good knowledge	222	52.5
Poor knowledge	201	47.5

ART, antiretroviral therapy; HBV, hepatitis B virus; HIV, human immune deficiency virus.

### The magnitude of condom usage

The overall prevalence of condom use among HIV-positive pregnant women was 24.8%, whereas only 14.2% (95% CI 10.9%, 17.5%) used condom consistently during sexual intercourse.

### Factors associated with consistent condom use

Bivariable and multivariable logistic regression analyses were done to identify the factors associated with the women’s consistent condom use. In bivariable logistic regression, women’s higher educational status, testing positive for HIV during pregnancy, disclosing their HIV status to sexual partner, having a CD4 count > 600 cells/mm, being positive for HBV, and having alive child had been associated with consistent condom use. However, on the multivariable logistic regression analysis, women have educational status of college and above studies, women testing positive for HIV during pregnancy, women disclosing their HIV status to sexual partner, and women having a CD4 count > 600 cells/mm were the factors associated with consistent condom use.

The odds of using condoms consistently were 6.33 times higher among women having educational status of college and above studies compared to women who could not write and read (AOR = 6.33, 95% CI 1.96, 20.42). Likewise, the odds of pregnant women’s consistent condom use among women whose CD4 count was > 600 cells/mm were 4.78 times higher than their counterparts (AOR = 4.78, 95% CI 2.08, 10.97).

This study found that the odds of pregnant women’s consistent condom use were 5.99 times higher among women testing positive for HIV during non-pregnancy compared with women testing positive for HIV during pregnancy (AOR = 5.99, 95% CI 3.01, 11.94).

Finally, women who disclosed their HIV status to sexual partner were 4.85 times more likely to report consistent condom use compared with women who did not disclose (AOR = 4.85, 95% CI 1.71, 13.71) (Table 3).

**TABLE 3:** Bivariable and multivariable logistic regression analysis of factors associated with consistent condom use among HIV-positive pregnant women in the Amhara Region Referral Hospitals, Ethiopia, 2020/2021 (*n* = 423).

Variables	Consistent condom use		COR (95%CI)	AOR (95%CI)
	Yes	No		
**Educational status of the mother**				
Cannot read and write	4	73	1	1
Can read and write	3	60	0.91 (0.18, 4.23)	1.04 (0.20, 5.31)
Primary (1–8)	7	59	2.16 (0.60,7.75)	2.66 (0.64, 11.03)
Secondary (9–12)	9	41	4.00 (1.16, 13.82)	3.10 (0.80, 11.99)
College and above	37	130	5.19 (1.78, 15.15)	6.33 (1.96, 20.42)*
**Number of alive children**				
None	14	140	1	1
1–5	46	223	2.06 (1.09, 3.89)	1.96 (0.92, 4.17)
**Tested positive for HIV**				
During pregnancy	20	293	1	1
Before pregnancy	40	70	8.37 (4.60, 15.20)	5.99 (3.01, 11.94)**
**CD4 cells/mm**				
≤ 600 cells/mm	9	202	1	1
> 600 cells/mm	51	161	7.11 (3.39, 14.87)	4.78 (2.08, 10.97)**
**Disclosure of HIV status to sexual partner**				
Yes	55	245	5.29 (2.06, 13.58)	4.85 (1.71, 13.71)*
No	5	118	1	1
**HBV**				
Positive	7	10	4.66 (1.70, 12.77)	3.93 (0.9, 13.10)
Negative	53	353	1	1

HBV, hepatitis B virus; HIV, human immune deficiency virus; AOR, adjusted odds ratio; COR, crude odds ratio; CI, confidence interval; 1, reference category.

*p < 0.05, **p ≤ 0.001.

## Discussion

Consistent condom use is a cornerstone in public health for the prevention of HIV and other sexually transmitted infections (STIs), especially among people with HIV/AIDS. The results of this study showed that the magnitude of consistent condom use among HIV-positive pregnant women was 14.2%. This finding is lower compared with previous studies conducted in Ethiopia (56.7%) (30), Nigeria (26.2%) (7), and Brazil (48%) (16).

The possible discrepancy from the Ethiopian study might be due to the difference in study population. The study participants in that study were among sexually active women who were on ART; those with HIV and pregnant women were not included. Being HIV-positive and pregnant at the same time might be a key factor for inconsistent condom use. The studies showed that being sexually active non-pregnant women was a determinant factor for consistent condom use (30, 31). This could be due to the fact that sexually active HIV-positive women use condoms to prevent unwanted pregnancy (30).

The possible difference from the study conducted in Brazil might be the variation in study setting and sociodemographic profile. The study participants in Brazil were in the third trimester of pregnant women, a clear difference of community level of understanding. Staying longer duration in prenatal clinics provides a good opportunity for effective promotion of condom use (32), which could result in a higher rate of consistent condom use. The study participants’ minimum level of education in Nigeria had been primary education (7). This could be the reason for the difference, and women clearly solicited behavior change through education (33).

This study found that women who had educational status of college and above studies were associated with women’s consistent condom use. The odds of using condoms consistently were 6.33 times higher among women having educational status of college and above studies compared to women who could not write and read. This study is consistent with previous studies (29, 34, 35). The plausible reason could be because the higher the education of an individual, the more likely an individual is to get the right information on the prevention and transmission of HIV/AIDS. Getting right information on HIV/AIDs prevention will enable the individual to use proper strategies to protect themselves from different infections (29). The other possible reason might be that women who have educational status of college and above studies might have higher socioeconomic status and more agency related to health behaviors compared to women who cannot read or write.

The odds of pregnant women’s consistent condom use whose CD4 count was > 600 cells/mm were 4.78 times more likely to report consistent condom use than their counterparts. This finding is consistent with a previous study (36). Higher CD4 counts may be due to new infection and taking ART for a long period of time also dramatically improves the number of CD4 counts. In this study, almost 74.7% of the participants were diagnosed for HIV before a year, which is a long time. The aim of using ART drug is to increase the CD4 count, and the longer the women received ART the more likely they are to use condoms consistently. The reason could be due to better HIV prevention programs/messages and counseling among ART-experienced women compared with those who were new to ART. Research has shown that the uptake of condom use increased with time, i.e., the longer the follow-up duration, the better the culture of condom use (37, 38). The transmission rate is minimal when the viral load is undetectable (39, 40). Overall, regardless of the viral load, it is better to use condom consistently during pregnancy.

Accordingly, this study found that the odds of pregnant women’s consistent condom use were 5.99 times higher among women testing positive for HIV during the non-pregnant state compared to women testing positive during pregnancy. As evidence shows, women new to their HIV status before pregnancy might stay a longer duration on ART care services (35). This could result in getting more health education from healthcare providers about HIV prevention programs and counseling compared to those who started ART recently. Women’s knowledge on their HIV status and consistent condom use increased with the duration of follow-up, and more than half (56.2%) of the study participants know they were positive for HIV within 2–3 years. This time is more likely the non-pregnant period for women who were the study participants in this study. This will have influenced their decision to use condom consistently during pregnancy (29, 35). The other reason might be that women enough time to think of the implications of HIV infection on their husbands as well as their unborn child. These all could be the reasons for using condom consistently than those recently testing positive. In this study, only 25.4% of the study participants knew their HIV status for less than a year, and this is one of the possible reasons why a long duration of follow-up could increase the odds of condom use. In addition, the likely reason might be that newly diagnosed pregnant women have been accredited to post-traumatic stress disorder following HIV diagnosis (41). This occurs in many women whose test being HIV-positive for the first time during pregnancy, resulting in depression. This could be an upsetting event of being pregnant and HIV-positive nearly around the same time (42). Therefore, they might not be emotionally ready to use condom due to the fear of negative consequences (29, 43, 44).

Disclosing their HIV status to their sexual partner was an important factor in women’s consistent condom use. This is consistent with a previous study (29). Telling how they feel about their sexual partners could be helpful and may be a useful source of support (45). Besides, joint discussion with sexual partners will help in obtaining ideas about the benefits of condom use in the prevention of HIV transmission, infection, and reinfection in couples to protect their unborn child from infections.

## Limitations of the study

Consistent condom use was measured based on self-reported information, which is affected by socially desirable bias because the topic is sensitive and results in over-reporting. In addition, the result did not show the real cause-and-effect relationship due to the nature of the study design.

## Conclusion

Consistent condom use among HIV-positive pregnant women in this study was low. Women having educational status of college and above studies, women testing positive for HIV during the non-pregnant state, women disclosing their HIV status to sexual partner, and women having a CD4 count > 600 cells/mm had better consistent condom use. Thus, the concerned bodies and relevant stakeholders better set educational strategies for better women’s educational status. Besides, more emphasis must be given to HIV-positive women on the health education and counseling service regarding HIV testing before pregnancy and the need for disclosing their HIV status to their sexual partners. Moreover, it is better to empower women to use condom. consistently during pregnancy.

## Data availability statement

The raw data supporting the conclusions of this article will be made available by the authors, without undue reservation.

## Ethics statement

The studies involving human participants were reviewed and approved by University of Gondar Institution Review Board (IRB). The patients/participants provided their written informed consent to participate in this study.

## Author contributions

NT was involved in the conception and design of the study, participated in data collection, analyzed the data, and drafted the manuscript. BK, BR, AK, TA, WT, MBA, KW, MYA, and HA approved the proposal with some revisions, participated in data analysis, wrote the manuscript, and revised subsequent drafts of the manuscript. All authors have read and approved the submitted version of the manuscript.

## Abbreviations

AIDS: acquired immune deficiency syndrome
AOR: adjusted odds ratio
ANC: antenatal care
ART: antiretroviral therapy
CI: confidence interval
COR: crude odds ratio
ETB: Ethiopian Birr
HIV: human immune deficiency virus
PLWHA: people living with acquired immune deficiency virus
PMTCT: prevention of mother to child transmission
MTCT: mother to child transmission
SPSS: statistical package for social science
STD: sexually transmitted diseases.
